# *Theragra chalcogramma* Hydrolysate, Rich in Gly-Leu-Pro-Ser-Tyr-Thr, Alleviates Photoaging via Modulating Deposition of Collagen Fibers and Restoration of Extracellular Components Matrix in SD Rats

**DOI:** 10.3390/md20040252

**Published:** 2022-04-01

**Authors:** Defeng Xu, Caihong Li, Mouming Zhao

**Affiliations:** 1 Guangdong Provincial Engineering Technology Research Center of Marine Food, Guangdong Provincial Key Laboratory of Aquatic Product Processing and Safety, College of Food Science and Technology, Guangdong Ocean University, Zhanjiang 524088, China; 2 Collaborative Innovation Center of Seafood Deep Processing, Dalian Polytechnic University, Dalian 116034, China; 3 School of Basic Medical Sciences, Guangdong Medical University, Dongguan 523808, China; 15916938195@163.com; 4 School of Food Science and Engineering, South China University of Technology, Guangzhou 510640, China

**Keywords:** photoaging, TCH, ECM metabolism, MMP-1, pathological restoration

## Abstract

Excessive exposure of the skin to ultraviolet irradiation induces skin photoaging, which seriously deteriorates the barrier functions of skin tissue, and even causes skin damages and diseases. Recently, dietary supplements from marine sources have been found to be useful in modulating skin functions and can be used to alleviate photoaging. Herein, the low-molecular-weight hydrolysates with a photoaging-protection effect were prepared by enzymatic hydrolysis from *Theragra chalcogramma* (TCH), and the potential mechanism were subsequently explored. The results revealed that TCH desirably improved the barrier functions of photoaged skin and stimulated the deposition of ECM components Col I, Hyp, and HA in the dermal layer. Histologically, TCH reduced the epidermal hyperplasia and restored the impaired architectures in a dose-dependent manner. Meanwhile, the activity of matrix metalloproteinase-1 (MMP-1) in photoaging skin was inhibited, and the expression levels of elastin and fibrillin-1 were elevated accordingly after TCH administration, and the significant improvements were observed at high-dose level (*p* < 0.05). Taken together, the efficacy of TCH against skin photoaging is highly associated with the regulation on ECM metabolism and the repairing of damaged mechanical structure.

## 1. Introduction

Excessive chronic exposure of skin to solar ultraviolet irradiation gradually induces photoaging, with morphological aggravation of roughness, deep wrinkles, and pigmentation in skin tissue. In addition, the degradation of barrier functions in photoaging skin were observed by the reduction of epidermal moisture and sebum contents, and elasticity decrease. Biochemically, photoaging is a progressive and complex process, during which the accumulated reactive oxygen species (ROS) induced by excessive UV irradiation mediate a series of cascading biochemical reactions, subsequently leading to skin damage and diseases, as well as the losses of skin aesthetics [1,2,3]. Various reports have revealed the upregulation of matrix metalloproteinases (MMPs), and MMP-1 is especially responsible for the destruction of collagen ingredients and triggers the photoaging process [4,5,6,7]. Recently, with the increasing enhancement of the populations’ aesthetic requirements, the prevention and cure for skin photoaging has received more attention, and the corresponding strategies for targeting inhibition of MMP-1 have been proved to be reasonable.

Currently, many approaches that intervene with the photoaging process have been developed, and the functional ingredients extracted from the animal or plant resources are drawing more attention. However, most of these ingredients are used as topical applications and must be absorbed into dermis tissue for alleviating the wrinkling [8]. Histologically, skin tissue contains epidermis, dermis, and hypodermis, among which the epidermis is essential for defensive purposes, and the defensive function of epidermis largely depends on the integrity of skin barrier architectures, conventionally described as the brick-and-mortar model. Owing to the compact and stacked architecture, however, stratum corneum strictly prevents the penetration of molecules more than 3000 Da or nanoparticles of >1000 nm in one dimension into skin tissue [9,10]. In addition, the active ingredients in most formulas are liable to be decomposed and even produce toxic substances for a long duration of UV exposure [11]. Therefore, oral administration of active ingredients from food-derived resource has received more attention for repairing the disorganized structure and restoring the barrier functions of photoaged skin.

In the first decades, many scientific reports have revealed the desirable efficacy of oral administration of vitamin C, vitamin E, flavonoids, polysaccharides, and peptides on the reduction of wrinkle reformation in photoaging skin [12,13,14,15,16]. Notably, food-derived peptides have been paid more attention for their potent biological activities, and many kinds of anti-aging peptides have been reported so far [17,18,19,20]. The peptides prepared with enzymatic hydrolysis from cod skin collagen exhibited significant photoaging protection by excellent inhibition on MMPs [6,7,12,16]. In addition, after oral administration of collagen peptide, skin damage, such as water loss, epidermal hyperplasia, and reduction of type I collagen (Col I) induced by UVB irradiation in hairless mice, was markedly attenuated [18,19]. Furthermore, the oral administration of collagen peptide, which is rich in VERISOL, significantly inhibited the formation of eyelid skin wrinkles and stimulated the biosynthesis of procollagen, elastin, and fibrillin in human skin [8]. Although many food-derived peptides with anti-photoaging properties have been reported, little information about the *Theragra chalcogramma* processing by-product was available until now. As a commercially important fish species in China and throughout the world, the biomass of processing by-products from *Theragra chalcogramma* is increasing, and only a few works in the literature about the peptides’ release from *Theragra chalcogramma* exist so far [21,22,23], and no information on the anti-photoaging effects of *Theragra chalcogramma* hydrolysates is available. Therefore, in the present study, we aimed to prepare the hydrolysate with potent photoaging prevention from the discarded industrial processing by-product of *Theragra chalcogramma* (TCH, rich in low-molecular-weight peptides) by enzymatic proteolysis, and then to further clarify the underlying mechanism. This investigation provides a solid foundation for the high-value utilization of frame protein from *Theragra chalcogramma* processing by-products.

## 2. Results and Discussion

### 2.1. Preparation of Low Molecular Weight TCH

**Figure 1:**
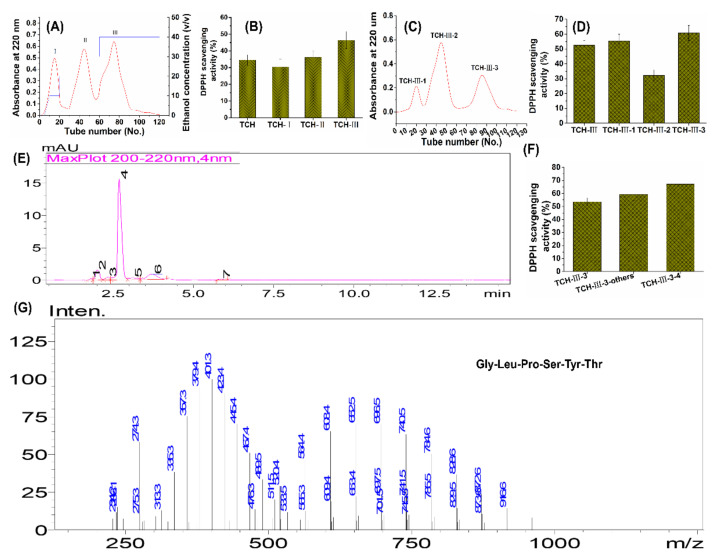
Preparation, isolation, and characterization of TCH with high antioxidant capacity. The low-molecular-weight hydrolysates in TCH were firstly isolated with SP-825 macroporous adsorption resin (**A**), and the hydroxyl radical scavenging activity of TCH and its three fractions was compared (**B**); then the fraction TCH- III with highest DPPH hydroxyl radical scavenging activity was subsequently isolated with Sephadex G-15 column (**C**) and fraction TCH-III-3 showed the highest activity among the three subfractions (**D**); next, fraction TCH-III-3 was further isolated with RP-HPLC (**E**) and TCH-III-3-4 exhibited the highest scavenging activity compared to other subfractions (**F**); eventually, the fraction TCH-III-3-4 was analyzed with LC–MS/MS, and the principal fragments were identified accordingly (**G**).

To obtain the functional fragments, we isolated the low-molecular-weight TCH and identified it subsequently. At first, the protein hydrolysate was filtered by using ultrafiltration membranes with 3 kDa molecular-weight cutoffs. To exclude the protein and polypeptides components in hydrolysate, only the fractions less than 3 kDa were collected and further isolated with SP-825 macroporous adsorption resin. As shown in Figure 1, among TCH and its three fractions, fraction TCH- III exhibited the highest DPPH hydroxyl radical scavenging activity and was then isolated with Sephadex G-15 column. Among the three subfractions, TCH-III-3 exhibited the highest scavenging capacity and was further isolated with RP-HPLC. Overall, TCH-III-3-4 illustrated the highest DPPH scavenging activity compared to the other subfractions, and the principal fragment, Gly-Leu-Pro-Ser-Tyr-Thr, was eventually identified with LC–MS/MS. Numerous reports have revealed that skin photoaging is initiated and accelerated by excessive ROS production [24,25,26]. Therefore, the antioxidative peptides with strong free-radical-scavenging capacity could eliminate the excessive ROS and exhibit considerable benefits against photoaging. Based on this close correlation between antioxidant and anti-photoaging, we inferred that TCH performs photoaging protection through the antioxidant capacity, and future work should be pay more attention to the relation between the structure of active peptides and anti-photoaging efficacy.

### 2.2. Effects of TCH on the Appearance and Body Weight in Photoaging Skin

**Figure 2:**
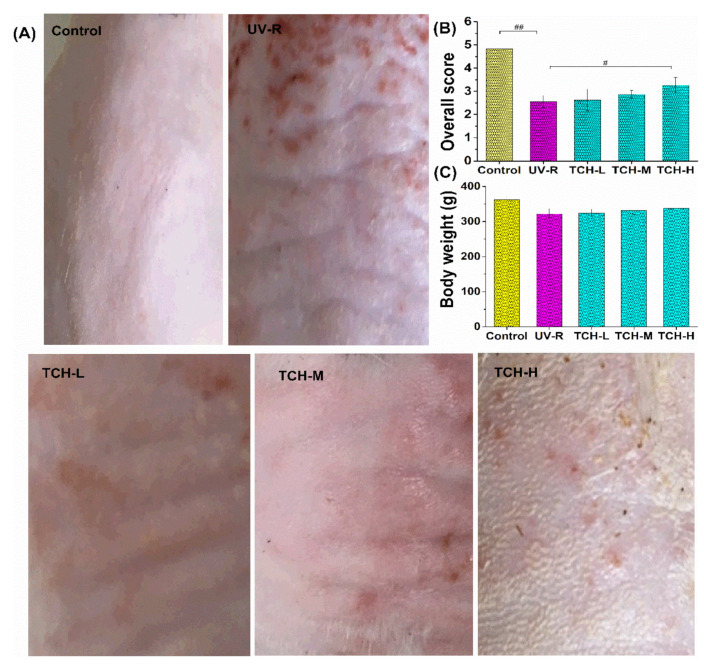
Effects of TCH administration on the physiological appearance (**A**), overall score (**B**), and body weight gains in photoaging SD rat (**C**). The # and ## indicate significant difference at the *p* < 0.05 and *p* < 0.01 level, respectively, when compared with that in the UV-R group.

The typical feature of photoaging is the degradation of macroscopic appearance. As shown in Figure 2A,B, the dorsal skin of rats in the normal group appeared to have a few shallow wrinkles, and the overall score was as high as (4.91 ± 0.21), whereas the deep wrinkles and leathery appearance were observed in the UV-R group, and the overall score significantly dropped to (2.22 ± 0.21) (*p* < 0.01). After intervention with TCH, this appearance degradation was obviously ameliorated, and the overall score increased significantly to (3.74 ± 0.61) in the TCH-H group (*p* < 0.05), demonstrating the high efficacy of TCH in mitigating the wrinkle formation. In addition, the slight improvement of TCH on the growth attribute was observed in photoaging rats (Figure 2C). In comparison with the body weight of rats in the normal control group, the values in the UV-R group obviously decreased, indicating the inhibition of excessive UV irradiation on growth. After the oral administration of TCH, this inhibition was alleviated to some extent, and this promotion of food-derived protein hydrolysate on animal growth has been demonstrated in many scientific reports [27,28]. In the present study, this similar tendency was further observed, and this alleviation might be contributed to its regulation on intestinal functions.

### 2.3. Effects of TCH on the Barrier Functions in Photoaging Skin of SD Rats

**Figure 3:**
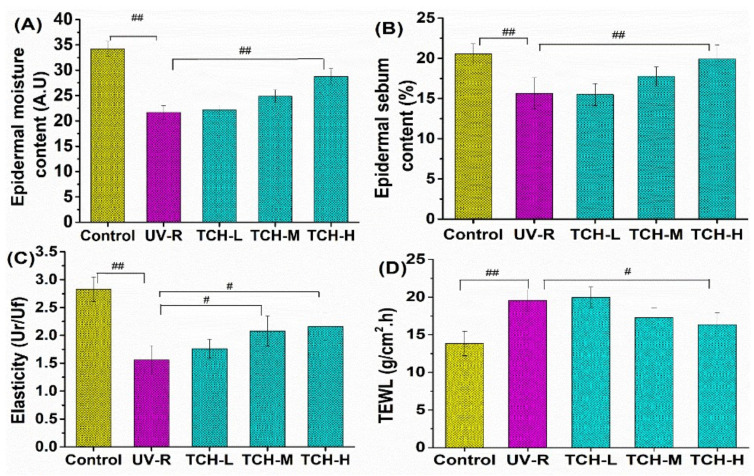
Effects of oral administration of TCH on the barrier functions in photoaging skin. (**A**) TCH increased the epidermal moisture content in UV-induced photoaging skin, (**B**) TCH increased the epidermal sebum content in UV-induced photoaging skin, (**C**) TCH enhanced the skin elasticity in UV-induced photoaging skin, and (**D**) TCH reduced the TEWL in UV-induced photoaging skin. The # and ## indicate significant differences at the *p* < 0.05 and *p* < 0.01 level, respectively, when compared with that in the UV-R group.

Barrier functions of skin tissue are the typical indicators to evaluate the photoaging status. As shown in Figure 3, the values of epidermal moisture (Figure 3A), sebum contents (Figure 3B), and elasticity (Figure 3C) of rats’ dorsal skin in the normal control group were (34.15 ± 1.19) AU, (20.98 ± 1.24)%, and (2.77 ± 0.65) Ur/UF, respectively, whereas all the corresponding values in the UV-R group significantly decreased (*p* < 0.01). On the contrary, the indicator of TEWL significantly increased (Figure 3D, *p* < 0.01). Obviously, these findings suggested the serious degradation in skin barrier functions owing to the chronic UV irradiation. With the intervention of TCH, the barrier functions were improved in a dose-dependent manner, demonstrating the modulation of TCH on barrier functions in photoaging skin.

### 2.4. Effects of TCH on the Pathological Impairments in Photoaging Skin

**Figure 4:**
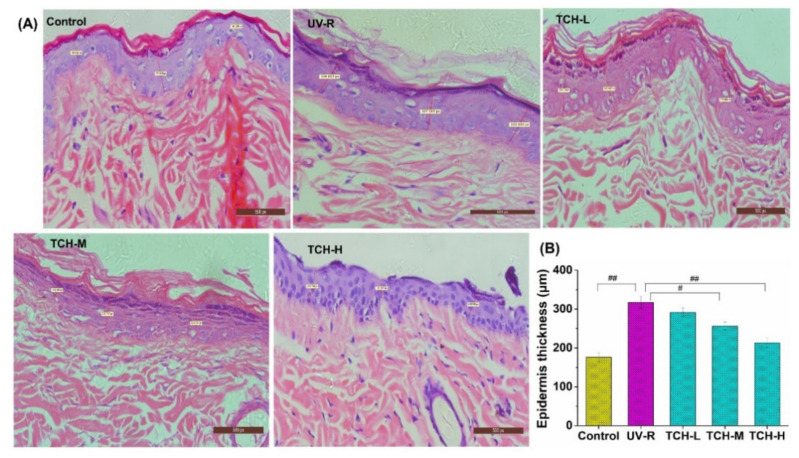
Effects of TCH administration on the histopathological abnormalities of epidermal sections in photoaging skin. (**A**) Representative photographs of sections by hematoxylin and eosin staining (200× magnification). (**B**) Effect of TCH on the epidermal thickness in photoaging skin. The # and ## indicate significant differences at the *p* < 0.05 and *p* < 0.01 level, respectively, when compared with that in the UV-R group.

The skin histopathology results can further explain the degradation of barrier functions in photoaged skin [6,29]. The improving effect of TCH on the disorganized structure of photoaged skin was histologically investigated. As shown in Figure 4A, the obvious pathological impairments, such as epidermal hyperplasia, keratinocytes edema, and fibroblasts loss, were observed in the UV-R group. Exactly, the epidermal thickness of dorsal skin significantly increased from (175.43 ± 12.12) μm in the normal control group to (317.26 ± 12.26) μm in the UV-R group (Figure 4B, *p* < 0.01). With the administration of TCH, the severe epidermal hyperplasia was gradually alleviated, and the value of epidermal thickness in the TCH-H group decreased to (213.26 ± 15.11) μm, significantly lower than that in the UV-R group (*p* < 0.05). The typical features of photoaged skin, such as epidermis thickening, dermis thinning, and basement membrane flattening, largely result from the degeneration and distortion of dermal fibrous components [30]. In the present study, the similar phenomena in epidermal deterioration were observed, and the macroscopic wrinkles in photoaged skin were markedly reduced after TCH administration, indicating the considerable alleviation of TCH in epidermal hyperplasia and damages in the mechanical structure.

**Figure 5:**
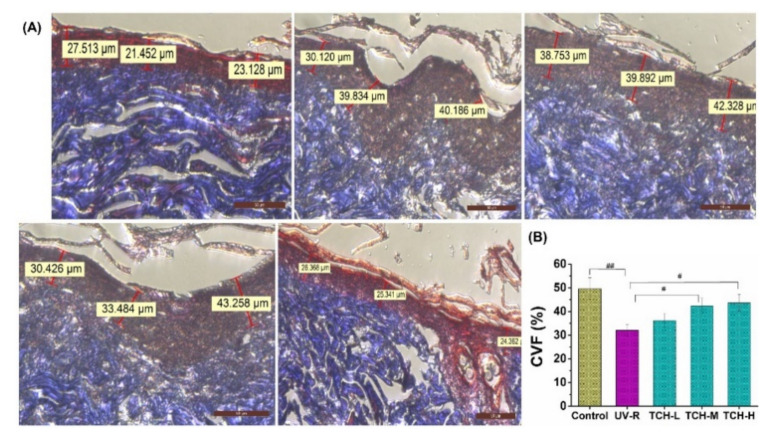
Effects of TCH administration on histopathological abnormalities of dermis sections in photoaging skin. (**A**) Representative photographs of sections by Masson staining (200× magnification). (**B**) Effect of TCH on the CVF in photoaging skin. The # and ## indicate significant differences at the *p* < 0.05 and *p* < 0.01 level, respectively, when compared with that in the UV-R group.

Furthermore, dermal collagen fibrils are regarded as the major components of structural proteins in skin tissue and the fragmentation of collagen fibrils is one of the prominent histopathological features of photoaging [30,31]. The result of Masson-trichrome staining showed the intact and waving arrangement of collagen fibril in the normal control group with the deep blue color of collagen fibers (Figure 5A). On the contrary, the waving arrangement of collagen fibril disappeared in the UV-R group, and the color of collagen fibers changed from the deep blue to light blue, indicating the substantial reduction of collagen density. With TCH administration, the loss of collagen fibril was gradually alleviated, and the waving arrangement structure was restored in a dose-dependent manner, suggesting the marked repairing effect of TCH on destroyed dermal structure. On the other hand, the value of collagen volume fraction (CVF) decreased significantly from (49.33 ± 1.25)% in the normal group to (32.18 ± 1.49)% in the UV-R group (*p* < 0.01, Figure 5B). After the intervention with TCH, the value increased in a dose-dependent manner, and a significant enhancement of (42.43 ± 1.69)% was observed in the TCH-H group (*p* < 0.05), revealing the substantial promotion of TCH on biosynthesis of collagen fibers and powerful repairing effect on the damaged dermal structure in photoaging skin.

### 2.5. Effect of TCH on ECM Components

**Figure 6:**
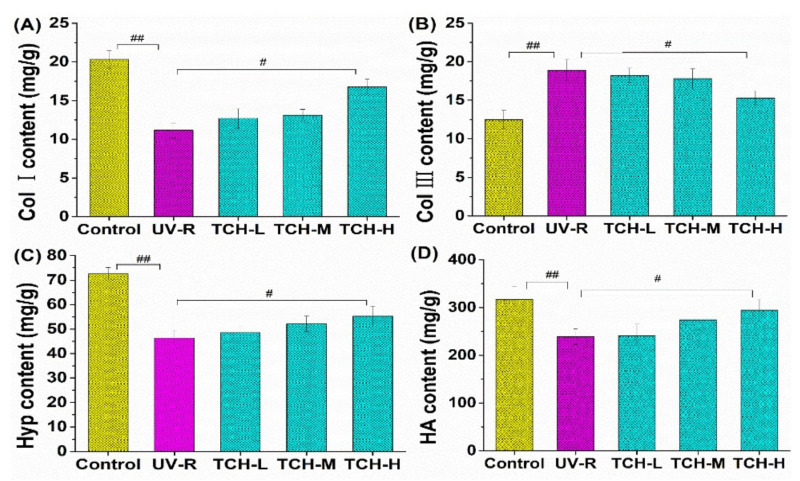
Effect of TCH administration on ECM components on the photoaging skin of SD rats. (**A**) Content of Col I, (**B**) content of Col III, (**C**) content of Hyp, and (**D**) content of HA. The # and ## indicate significant differences at the *p* < 0.05 and *p* < 0.01 level, respectively, when compared with that in the UV-R group.

ECM is the material base for maintaining skin barrier functions, and collagen fibrils composed the principal parts of ECM. As the most abundant structural protein in skin connective tissue, collagen is responsible for providing skin with strength and resiliency [32,33,34,35]. Among the collagen fibers, Col I and III are highly associated with skin photoaging, in which Col I content negatively affects the photoaging process, whereas the content of Col III exhibits a positive effect on photoaging. As shown in Figure 6A, the Col I content significantly decreased from (22.03 ± 0.25) mg/g in the control group to (12.08 ± 0.16) mg/g in the UV-R group (*p* < 0.01). Compared with that in the UV-R group, the Col I content increased in a dose-dependent manner after TCH oral intervention, and the significant enhancement was found at a high dose level (*p* < 0.05). As for Col III, UV-R stimulated its significant high expression, and the oral administration of TCH obviously inhibited this expression (Figure 6B).

In addition, as the primary and unique component of collagen fibril, Hyp amounts to ~13% of the total collagen in skin tissue and thus can be used as an indicator to evaluate the photoaging status of skin [36,37,38,39]. Figure 6C illustrated the promotion of TCH on Hyp production in photoaging skin. UV irradiation significantly reduced the content of Hyp, in which the content decreased from (71.49 ± 3.15) mg/g in the normal control group to (43.32 ± 3.51) mg/g in the UV-R group (*p* < 0.01). After the oral administration of TCH, the Hyp content gradually increased and significantly increased to (58.38 ± 3.56) mg/g in the TCH-H group (*p* < 0.05). Moreover, HA plays important roles not only in maintaining skin moisture and structure, but also in promoting skin regeneration, enhancing skin elasticity, and degrading skin free radicals [38]. As shown in Figure 6D, the HA content significantly dropped from (469.14 ± 20.25) mg/g in normal control group to (211.67 ± 18.64) mg/g in the model group (*p* < 0.01). The obvious enhancement, however, was observed when TCH was administrated, and a significant elevation to (422.35 ± 13.21) mg/g was determined in the TCH-H group (*p* < 0.01). Taken together, the contents of the main components in ECM decreased significantly after UV irradiation, and this degradation was substantially alleviated after the oral administration of TCH, demonstrating the potent attenuation of TCH against skin photoaging by the favorable regulation of ECM components’ biosynthesis.

### 2.6. Effect of TCH on the Level of Elastin and Fibrillin-1

**Figure 7:**
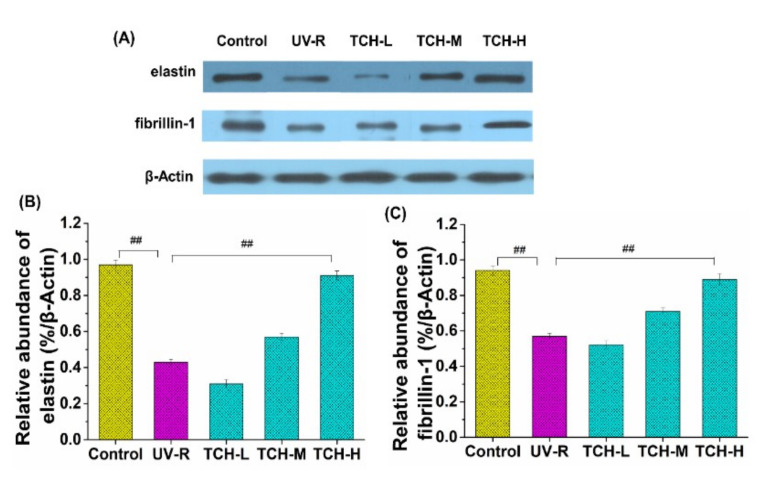
Effects of TCH on the expression levels of elastin and fibrillin-1 in the dorsal skin of SD rats. (**A**) Images of Western blotting of elastin and fibrillin-1 proteins. (**B**) Signal intensities were quantified and normalized to the corresponding value of β-actin and the quantified results of relative abundance of elastin. (**C**) The quantified results of relative abundance of fibrillin-1. The ## indicates significant differences at the *p* < 0.01 level, when compared with that in the UV-R group.

As the principal structural protein in ECM, elastin plays a vital role in maintaining the stretching and recoiling properties of skin tissue. Meanwhile, the microfibril component fibrillin is closely connected with elastin and assists in the formation, assembly, and stabilization of elastin [40]. Therefore, the expression levels of elastin and fibrillin are crucial for the dermal integrity and elasticity of skin tissue. As shown in Figure 7, UV irradiation significantly reduced the expression level of elastin (Figure 7A) and fibrillin-1 (Figure 7B), with decreases of 87.6 and 40% in comparison with those in the normal control group, respectively. With administration of TCH, however, the enhancement on elastin and fibrillin-1 expression was observed, and a significant elevation was observed for the TCH-H group (*p* < 0.05), indicating the considerable upregulation of TCH on the elastin and fibrillin-1 expression in photoaging skin.

### 2.7. Effect of TCH on the MMP-1 Activity in Photoaging Skin

**Figure 8:**
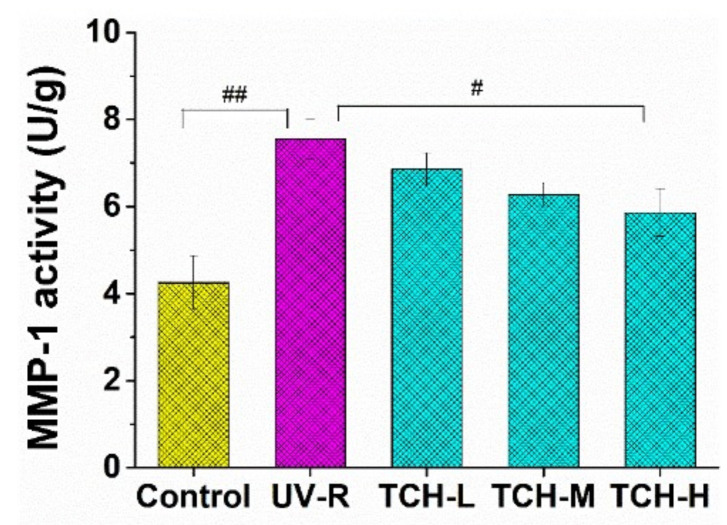
Effect of TCH administration on MMP-1 activity in photoaging skin of SD rats. The # and ## indicate significant differences at *p* < 0.05 and *p* < 0.01 level, respectively, when compared with that in UV-R group.

Generally, MMPs mediate the process of collagen destruction, and considerable research studies have demonstrated the elevation of MMPs in photoaging skin and their involvement in decomposition of connective tissue [41,42]. Among the MMPs, MMP-1 is the critical driving force for the degradation of collagen fibril and intact structure. It is therefore a crucial step to reduce the MMP-1 expression for inhibiting the formation of coarse wrinkles. As illustrated in Figure 8, the MMP-1 activity of (6.74 ± 0.23) U/g in UV-R group was significantly higher than that of (3.55 ± 0.19) U/g in the normal control group (*p* < 0.05), indicating the activation of MMP-1. With oral administration of TCH, the MMP-1 activities were inhibited in a dose-dependent manner, and the significant inhibition was observed in the TCH-H group (*p* < 0.05). In view of the close correlation between activation of MMPs and collagen decomposition, the protective effect of TCH on collagen degradation may result from the powerful inhibition of MMP-1 activity in photoaging skin.

**Figure 9:**
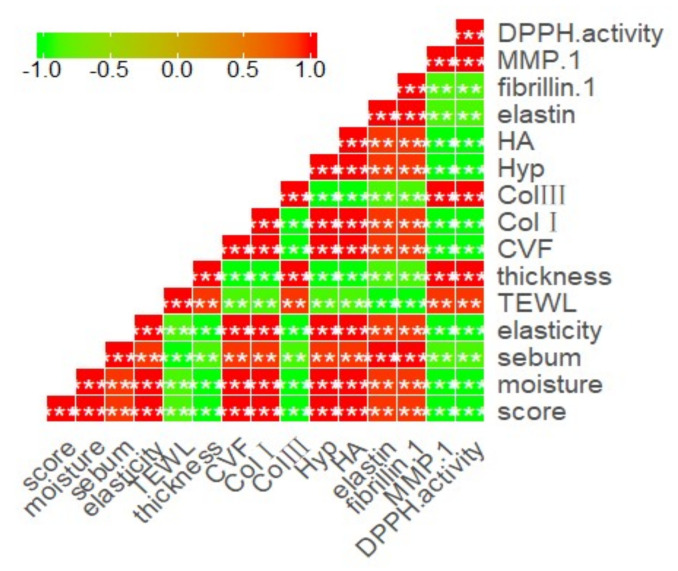
Correlations between DPPH activity and the skin properties.

To further clarify the association of antioxidation of TCH with photoaging properties, we analyzed the correlation between DPPH activity with the determined indicators. As illustrated in Figure 9, there are significantly positive correlations between DPPH activity and the indicators of MMP-1 activity, contents of Col III, epidermal thickness, and TEWL, whilst a significant negative correlation was observed between DPPH activity and other indicators. These data strongly revealed the significance of antioxidative activity of TCH in photoaging prevention.

**Figure 10:**
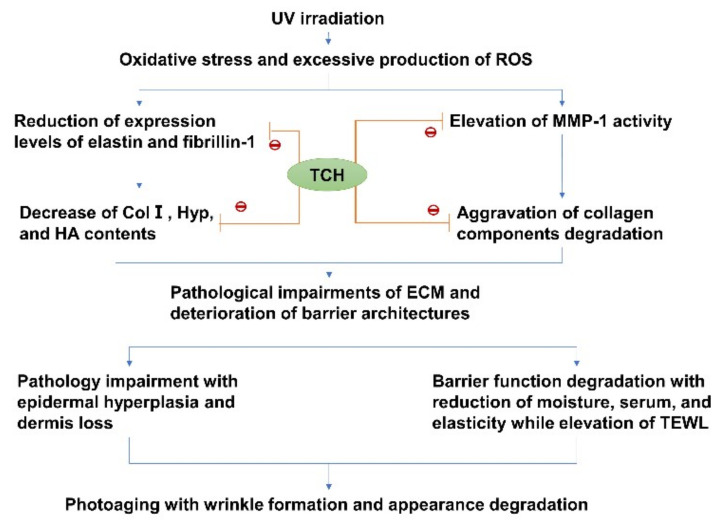
Potential cascading mechanism of TCH against photoaging.

Moreover, a considerable number of works from the literature have reported that the UV irradiation induces excessive oxidative stress and eventually leads to photoaging [24,25,26]. Therefore, the antioxidative peptides with potent free-radical-scavenging capacity could be used to inhibit photoaging progression. In our present study, TCH was prepared by enzymatic hydrolysis, exhibited excellent free radical-scavenging capacity, and considerably ameliorated the barrier functions in photoaging skin. Moreover, TCH was beneficial to restore the damaged ECM architecture, stimulated the deposition of collagens, and inhibited the MMP-1 activity. Taken together, this study clearly suggests that a certain dose of TCH oral administration could increase the thickness of dermal collagen, make collagen fibers more compact, and improve the elasticity of photoaged skin. Overall, the underlying mechanical mechanism of TCH against photoaging was illustrated in Figure 10. The future study should pay more attention to the relation between the structure of active peptides in TCH and anti-photoaging efficiency.

## 3. Materials and Methods

### 3.1. Preparation of TCH

*Theragra chalcogramma* was purchased from a local supermarket in Zhanjiang City, China. After elimination of flesh tissue, the hide and frame were chopped into small pieces and mixed with distilled water, with a ratio of hide to water of 1:3 (weight: volume), following with autoclavation at high temperature and pressure (121 °C and 1.5 kgf/cm^2^) to extract the proteins. After thorough liquefaction, the extract was filtered by using a mesh sieve and then hydrolyzed with Foodpro trypsin at an enzyme/substrate ratio (E/S) of 0.3:100, 45 °C, and pH 7.0, for 4 h. After 4 h of incubation, hydrolysis reactions were quenched by heating at 95 °C for 10 min, and the hydrolysates were centrifuged at 12,000× g and 4 °C for 15 min, and then the supernatant was filtered through the 3 kDa MWCO ultrafiltration membrane to acquire the short-chain peptides (<3 kDa). The peptide content was detected according to Kim et al. (2018) [18]. By virtue of the quick, convenient, and efficient advantages over other methods in evaluating the antioxidant activities of protein hydrolysates, the radical scavenging capacity of TCH on 1,1-diphenyl-2-picrylhydrazyl (DPPH (Sigma, D21140-0)) was used to evaluate the antioxidant activity of each fraction. The isolation of hydrolysate for highest antioxidant fraction was subsequently conducted with SP-825 macroporous adsorption resin (Lvbeicao Sci. & Tech. Co., Ltd. Beijing, China), gel permeation chromatography (Sephadex G-15 column, 4 × 65 cm^2^) and medium pressure liquid chromatography preparation system (AKTA Avant 25, GE Healthcare, Pittsburgh, PA, USA). At last, the fraction with the highest antioxidant activity was identified by UPLC–ESI–MS/MS (Bruker Daltonics Inc., Billerica, MA, USA) in the positive electrospray ionization (ESI+) mode via the electrospray interface.

### 3.2. Materials

The animal feed and shavings pad were from Xinhua Experimental Animal Factory (Huadu District, Guangzhou, China). The depilating agent of sodium sulfide was purchased from Hong Ming Chemical Reagent Co., ltd. (Jining, China). The commercial kits for detecting type I and III collagen (Col I and Col III), hydroxyproline (Hyp), hyaluronic acid (HA), hematoxylin–eosin (HE), and Masson staining were purchased from Jiancheng Biological Technology Co., Ltd. (Nanjing, China).

### 3.3. Rats

Sixty healthy female SD rats with the weight (180 ± 11) g and production license number of SCXK (Guangdong) 2019-0034, quality certificate number of No. 44005800002875, were purchased from the experimental animal center in Guangzhou University of Chinese Medicine. The rats (three in each cage) were accommodated in a clean-grade environment of 25 °C, 70% humidity, and natural light/dark cycle. All animal experimental procedures were in accordance with the Guidelines for Care and Use of Laboratory Animals of Guangdong Ocean University, and the experiments were approved by the Animal Ethics Committee of Guangdong Ocean University (Approval # SYXK 2019-0053).

### 3.4. Diet

After 2 weeks of environmental acclimation, the rats were randomly divided into 5 groups (n = 12): (1) normal control group, without exposure to UV irradiation and free access to the drinking water, control; (2) photoaging model group, with exposure to UV irradiation and free access to the drinking water, UV-R; (3) TCH oral administration group at low dosage, with exposure to UV irradiation and free access to the drinking water containing 0.32 g/L of TCH, TCH-L; (4) TCH oral administration group at medium dosage, with exposure to UV irradiation and free access to the drinking water containing 0.96 g/L of TCH, TCH-M; and (5) TCH oral administration group at high dosage, with exposure to UV irradiation and free access to the drinking water containing 2.88 g/L of TCH, TCH-H.

### 3.5. Establishment of Photoaging Model

Our photoaging model was established by referring to the description by Feng et al. (2014) and our previous report [5,43]. Briefly, an area of about 5 × 5 cm^2^ in both sides of the dorsal spine of rats was marked, and the hair within this area was removed with an electric clipper and 7% Na2S solution for 10 min. Then the SD rats, except those in the control group, were fixed on a self-made panel and exposed to the UV lamps simulating the natural solar violet irradiation, in which a UVA spectrum was provided with two lamps of Philips 340-40WT12-G13, and UVB was provided with three lamps of Philips 313-40WT12-G13, and the irradiation intensity was monitored by a radiometer from Beijing Normal University of Photoelectric Instrument Factory, Beijing, China. UV irradiation exposure was performed every two days, and each irradiation time lasted for 15 min during the first week, 20 min during the second week, and 30 min during the third week and lasting to the end of experiment, respectively. With consecutive UV radiation for 18 weeks, the obvious photoaging morphology in the rat skin of the UV-R group was observed and suggested the establishment of photoaging model. At this point, the total amounts of UV-A and UV-B reached 155.79 and 84.66 mW/cm^2^, respectively.

### 3.6. Measurement of Skin Morphology and Barrier Functions

The appearance and overall score were evaluated by a sensory panel, as described by Inomata et al. (2003) [44]. The epidermal moisture and sebum content, and the elasticity in the depilation area were measured by using an FC1502 Facecaie Skin Analyzer (Shenzhen Kier electronic apparatus factory, Shenzhen, China) according to the instrument’s specification. TEWL was measured quantitatively by using Tewameter (TM300, Courage + Khazaka, Cologne, Germany), and the value was automatically expressed as g/m^2^ h.

### 3.7. Determination of ECM Components and MMP-1 Activity

At the end of the final UV exposure, the rats were sacrificed by being anesthetized with diethyl ether, and the biopsies samples of about 1.50 g were obtained from the dorsal skin. After we removed the subcutaneous fat in the connective tissue, 10% skin tissue homogenate was prepared with a homogenizer (Silent Crusher M, Heidolph, Schwa Bach, Germany). The contents of Hyp, HA, Col I and III, and MMP-1 activity were assayed by commercial kits, according to the manufacturer’s protocol.

### 3.8. Western Blotting of Elastin and Fibrillin-1

Elastin and fibrillin-1 were assayed with Western blotting, referring to our previous report [43]. Briefly, the homogenized proteins were separated by sodium dodecyl sulfate–polyacrylamide gel (SDS–PAGE), as a routine process. The separated proteins were transferred to a nitrocellulose membrane (Amersham Pharmacia Biotech, Buckinghamshire, UK) with Bio-Rad Mini-Protean II apparatus (Bio-Rad Laboratories, Carlsbad, CA, USA). After through blocking of the membrane with defatted milk, the blots in the membrane were incubated with the primary antibody of antirat elastin or antirat fibrillin-1 overnight at 4 °C. Then, after incubation with the secondary antibody, the protein level was visualized by using an ECL reagent (Fujifilm, LAS-4000, Tokyo, Japan). The bands were scanned and quantified by measuring the optical densities, using the VIpro Platinum 1.1 software package (Version 12.9, UVItec, UK). With β-actin as the internal control for data normalization, the signal intensities of protein bands were quantified accordingly.

### 3.9. Histological Evaluation

After being embedded in paraffin, 6 μm of skin tissues were cut and stained with hematoxylin and eosin (H&E), as described by Takeuchi et al. (2010) [45]. The collagen deposition was stained with Masson’s trichrome staining procedure described by Yu et al. (2016) [46]. Images were visualized with a light microscope (Olympus BX51, Olympus Co. Ltd. (Shanghai, China)) and digital imaging system (Olympus DP71, Olympus Co. Ltd. (Shanghai, China)). The collagen volume fraction (CVF) was calculated as the ratio of collagen area to total area.

### 3.10. Statistical Analysis

The data were expressed as means ± standard deviation (SD) and were analyzed by SPSS 16.0. The significant differences between groups were determined by one-way ANOVA. Pairwise comparing was conducted if the variance was equal, and then the data were compared through the Least-Significant Difference (LSD) method, or conducted by Dunnett’s *t*-test. A *p* < 0.05 was used as the criterion for statistical significance.

## 4. Conclusions

The low-molecular-weight TCH, rich in fragment Gly-Leu-Pro-Ser-Tyr-Thr, was prepared by enzymatic hydrolysis and significantly improved the barrier functions in photoaging skin. Moreover, the underlying mechanical mechanism of TCH against photoaging lies in the modulation of ECM metabolism and restoration of pathological architectures. Exactly, the contents of Col I, Hyp, and HA and expression levels of elastin and fibrillin were elevated significantly, whereas the content of Col III and MMP-1 activity were reduced accordingly (*p* < 0.05 or 0.01). Furthermore, the correlation analysis verified the high association of antioxidative capacity of TCH with the photoaging prevention effects. This study reveals that TCH can effectively alleviate the detrimental effects of UV irradiation and has potential as an agent for anti-photoaging foods development.

## Data Availability

The raw/processed data required to reproduce these findings cannot be shared at this time, as the data also form part of an ongoing study.
